# A randomized controlled study of a social skills training for preadolescent children with autism spectrum disorders: generalization of skills by training parents and teachers?

**DOI:** 10.1186/1471-244X-14-189

**Published:** 2014-07-02

**Authors:** Vera Dekker, Maaike H Nauta, Erik J Mulder, Marieke E Timmerman, Annelies de Bildt

**Affiliations:** 1 Department of Child and Adolescent Psychiatry, University of Groningen, University Medical Center Groningen, Accare Groningen, Hanzeplein 1, 9700 RB, Groningen, The Netherlands; 2 Department of Clinical Psychology, University of Groningen, Grote Kruisstraat 2/1, 9712 TS Groningen, The Netherlands

**Keywords:** Social skills training, Autism spectrum disorder, RCT, Primary school, Treatment efficacy

## Abstract

**Background:**

Social skills training (SST) is a common intervention for children with autism spectrum disorders (ASDs) to improve their social and communication skills. Despite the fact that SSTs are often applied in clinical practice, the evidence for the effectiveness of these trainings for children with ASD is inconclusive. Moreover, long term outcome and generalization of learned skills are little evaluated. Additionally, there is no research on the influence of involvement of parents and teachers on effectiveness of SST and on the generalization of learned social skills to daily life. We expect parent and teacher involvement in SST to enhance treatment efficacy and to facilitate generalization of learned skills to daily life.

**Method/Design:**

In a randomized controlled trial (RCT) with three conditions, 120 participants with ASD at the end of primary school (10–12 years of calendar age) have been randomized to SST, SST-PTI (SST with Parent & Teacher Involvement), or care-as-usual. The SST consists of 18 group sessions of 1.5 hours for the children. In the SST-PTI condition, parents additionally participate in 8 parent sessions and parents and teachers are actively involved in homework assignments. Assessment takes place at three moments: before and immediately after the intervention period and at 6 months follow-up. Primary outcome is socialization, as an aspect of adaptive functioning. Secondary outcomes focus on specific social skills children learn during SST and on more general social skills pertaining to home and community settings from a multi-informant perspective. Additionally, possible predictors of treatment outcome will be assessed.

**Discussion:**

The current study is an RCT study evaluating SST in a large sample of Dutch children with ASD in a specific age range (10–12 years). Strengths of the study are the use of one manualized protocol, application of standardized and internationally used rating instruments, use of multiple raters, investigation of generalization of learned skills to daily life, and the evaluation of efficacy in the longer term by follow-up measures at 6 months after the end of training.

**Trial registration:**

NTR2405

## Background

Autism spectrum disorders (ASDs) are defined in the Diagnostic and Statistical Manual of Mental Disorders, 5th Edition (DSM-5) as a neurodevelopmental disorder and are characterized by severe and pervasive impairment in two developmental areas: limitations in social communication and the presence of restricted, repetitive behaviors [1]. The Social Communication domain as defined in the DSM-5 contains symptoms from the Social and Communication domain which were separate domains in the DSM-IV-TR. In the DSM-IV-TR, classifications within the autism spectrum were separated as well (i.e. autistic disorder [AD], Rett’s disorder, childhood disintegrative disorder [CDD], Asperger’s disorder and pervasive developmental disorder- not otherwise specified [PDD-NOS; [2]]), whereas the DSM-5 no longer contains specific ASD classifications. The symptoms are now seen as a continuum, ranging from mild to severe expression [3].

This revised perspective on ASDs reflects empirical research into the underlying factors for ASDs [3] and is in line with clinical presentation in daily practice. ASDs typically emerge during young childhood and they persist throughout the lifespan. The etiology of ASDs remains unclear. There is a strong involvement of genetic factors in ASD [4]. The prevalence of ASDs is estimated at 1 per 150 children and adolescents [5], with the ratio of boys to girls being 4:1.

Children with ASDs lack the behavioral repertoire that is necessary to interact with others. Deficits vary over children with ASDs and may include lack of orientation towards social stimuli, inadequate use of eye contact, no or inadequate initiation of social interaction, difficulties in interpreting verbal and nonverbal social cues, inappropriate emotional responses, and lack of empathy for others’ distress or emotions [6]. Besides variation between individuals with ASDs, the social communication within children with ASDs varies over time as well. Although limitations in the communication domain are the core symptoms of ASDs, this does not mean that there can be no development in this area at all [7]. However, learning and implementation of the behaviors necessary for social interaction may always remain harder, go slower, and be less automatic for children with ASDs than for other children [8,9].

At least part of the children with ASDs has a desire for more peer social interaction [7], but they do not act in the right way to generate fluent social interaction with peers. Children with ASDs often show substantial social relational problems when compared to typically developing children, because they have difficulty sharing affective experiences or understanding the perspective of others, which is important for social reciprocity and the development of friendships [6]. Additionally, parents and teachers have reported larger deficiencies in cooperation, assertion, and self-control than a matched group of their typically developing peers [6]. This may result in an increased risk for peer rejection and social isolation.

The social problems encountered already early in life may have serious consequences, often leading to major deviances in development as compared to typical development. From several studies it is known that social problems may also affect the achievement of normal developmental milestones and the establishment of satisfying peer and familial relationships [6,10]. Further on, social deficits continue to negatively impact social and occupational functioning into adulthood. Adults with ASDs or high-functioning autism (HFA) are much more likely to be un(der)employed than the general population and are much less likely to have satisfying social relationships [6]. Apart from the consequences for development in general, social skills deficits predict specific problems later in development such as mood and anxiety problems [7].

There are different types of interventions for individuals with ASDs. Most psychological interventions are developed from a behavioral perspective [11]. Because of the importance of the social communication skills in ASDs, treatment has focused on improving these skills. Over the last decades, several methods have been developed with that aim, e.g. social stories [12], peer-mediated interventions [13], parents-assisted interventions [9], computer-based interventions [14], and social skills groups [15].

Specific group-based social skills training (SST) has become an important part of treatment for children with ASDs in clinical practice and in schools [16,17]. An SST is a child specific intervention based on behavioral and social learning, during which children are taught specific skills, as for instance making eye contact, initiating a conversation, and cooperation [7]. SST is supposed to help children develop those skills that are most severely affected and at the same time most crucial for developing relationships with others. For children with social phobia or specific learning disabilities, SST has been shown to be an effective treatment [18,19]. However, the evidence for the effectiveness for SST for children with ASDs seems inconclusive [6,7,20,21].

The studies into SSTs so far show design limitations with respect to lack of a control group and randomized assignment, lack of adequate measurement of social skills and deficits associated with ASD, limited sample size, not using multiple informants, not including follow-up measurements, and lack of assessment of generalization of learned skills in daily life. Moreover, most of the studies have been performed in the US [6,7], except Yoo and colleagues [22].

In a recent meta-analysis, Reichow, Steiner & Volkmar [23] found only five studies, including a total of 196 participants, that were based on a randomized controlled trial design (RCT). All five studies included a control group that did not receive an intervention for social skills. They included studies until December 2011 in their analysis, all performed in the US. To the best of our knowledge, only one RCT study into group SST for children with ASDs has been published since, that included a randomized control group that did not receive an intervention for social skills. Additionally, this was the first study outside the US [22].

A summary of study characteristics and outcome measures of these studies is shown in Table 1. As can be seen in Table 1, the age ranges were broad (ages of included children 6–17 years). Setting, frequency, and duration of the intervention also varied between the studies. One study investigated an intensive five-week summer intervention with five 70-minute treatment cycles every day [21] and the other studies had weekly training sessions of 60–90 minutes [8,9,13,16,22]. The protocol underlying the SSTs differed between the studies and involvement of parents or peers varied as well. Involvement of teachers was scanty, if at all. In two studies [9,13] peers were involved in the sessions of the SST and in four studies the parents were involved in the training [8,9,16,21,22]. Based on their meta-analysis, Reichow, Steiner & Volkmar [23] concluded that no firm conclusions can be drawn on the efficacy of SSTs for ASDs with respect to improving social competence, social communication, emotion recognition, and quality of life for children with ASDs, due to all the differences between the studies and the outcomes. In more detail: In three studies the children in the SST condition improved significantly (small-medium effect size) compared to the control condition. In the two other studies, no difference was found between the treatment and the control groups. More information on the conclusions of the separate studies is provided in the last column of Table 1.

**Table 1:** Characteristics of published RCTs with control condition on SSTs in children with ASDs

**Auteur**	**N (INT/CON)**	**In-/exclusion criteria**	**Intervention**	**Follow-up**	**Outcome measures**	**Conclusion (pre/post treatment)**		
Frankel et al. (2010) [9]	76 randomized, 68 completed training (35/33)	ADOS/ADI-R ASD	Parent-assisted Children’s Friendship Training (CFT).	Only intervention group after 3 months	- Parent			
		2^nd^-5^th^ grade regular classroom			○ QPQ (Ho, Gu, Con, Eng, Dis)	*p <* .0001 (Ho, Dis)		
		VIQ > 60	12 weekly sessions of 60 minutes with concurrent sessions for child and parents. Class size was usually 10, with no more than 4 children with ASD.		○ SSRS social skills (As, SC)	*p < .*05 (SC)		
		Knowing rules of board and school yard games						
					○ SSRS problem behavior (In, Ex)	ns		
		Able to switch topics in conversation			- Child			
					○ PHS	*p* < .025		
		Excl: psychotropic medication			○ The Loneliness Scale	*p* < .025		
		Thought disorder			- Teacher			
		Clinical seizure disorder, gross neurologic disease or other medical disorder			○ PEI (Wi, Ag)	ns		
Laugeson et al. (2009) [8]	33 (17/16)	Clin dx ASD	Program for the Education and Enrichment of Relational Skills (PEERS).12 weekly sessions of 90 minutes with concurrent sessions for child and parents.	No	- Parent			
		Age 13–17			○ SSRS social skills	*p* < .05		
		IQ > 70			○ SSRS problem behavior	ns		
		English fluency (child and parent)			○ QPQ (Ho, Gu, Con)	ns		
					- Teens			
		Parent want to participate			○ QPQ (Ho, Gu, Con)	*p* < .025 (Ho)		
					○ TASSK	*p* < .0001		
		Social problems			○ FQS	*p* < .05		
					- Teacher (n = 13)			
		Excl: history of major mental illness			○ SSRS social skills	ns		
		Hearing, visual or physical impairments			○ SSRS problem behavior	ns		
Koenig et al. (2010) [13]	44 (25/19)	Clin dx ASD	16 weekly sessions of 75 minutes; 4 to 5 children/2 peer/ 2 licensed clinicians.	No	- Parent			
		ADOS/SCQ/PDD-BI score ASD			○ CGI – improvement	*p* = .001		
		Age 8–11			○ SCI (PSI, SI)	ns		
		IQ > 70						
		Excl: need for different treatment						
		ABC irritability > 18						
		CSI clinically						
Lopata et al. (2010) [21]	36 (18/18)	Clin dx HFASD	Summer training program.5 weeks intervention with Five daily 70-minute treatment cycles every day; 3 therapists/6 children.	No	- Parent			
		Age 7–12			○ ASC	*p* = .006 *d* = .584		
					○ SRS	*p* = .003 *d* = .625		
		IQ > 70			○ BASC-2-PRS – withdrawal	*p* < .001 *d* = 1.055		
		VCI/PRI > 80			○ BASC-2-PRS – social skills	ns *d* = .365		
		Expressive language score > 80			- Child			
					○ SKA	*p* < .001 *d* = 1.272		
					○ DANVA-2 (CF)	ns *d* = .532		
Solomon et al. (2004) [16]	18 (9/9)	Clin dx ASD	The Social Adjustment Enhancement Curriculum.20 weekly sessions of 90 minutes with concurrent sessions for child and parents; 3 therapists/5 children.	No	- Child			
		ADOS ASD, ADI-R AD			○ DANVA-2 (AF, CF)	*p* < .05 (AF, CF)		
		Also met DSM-IV criteria of ASD based on a clinical interview			○ Strange Stories Task	ns		
					○ Faux Pas Stories Task	ns		
		Age 8–12			○ TOPS-ER	*p* < .05		
		IQ > 75						
		Able to pass first theory of mind task						
		Excl: serious conduct problems						
Yoo et al. (2014) [22]	55(28/27)	Clin dx ASD	The PEERS Treatment Manual.14 weekly sessions of 90 minutes with concurrent sessions for child and parents.	Only intervention group after 3 months	- Child	I	II	III
		Age 12–18			○ TASSK-R	p < .01	p < .01	p = .01
					○ QPQ (Gu, Con)	ns	ns	ns
					○ QPQ (Ho)	ns	ns	p = .04
		School 6^th^ grade elementary school to 3^rd^ grade high school			○ K-SSRS (As, Co, Em, SC, T)	ns	ns	ns
					○ CDI	p = .04	p = .03	ns
					○ STAIC-T	ns	ns	ns
					○ STAIC-S	ns	ns	ns
		Social difficulties			○ ADOS (lc-a, lc-t)	p = .01	p < .01	p < .01
		VIQ ≥ 65			○ ADOS (rsi-a, rsi-t)	p < .01	p < .01	p < .01
					- Parents			
		Substantial treatment motivation			○ SCQ	ns	ns	ns
					○ SRS	ns	ns	ns
					○ QPQ (Gu, Con)	ns	ns	ns
		No history of major mental illness			○ QPQ (Ho)	ns	p = .03	p = .03
		No current problems with aggressive behavior or severe oppositional tendency			○ ASDS (L, SI, BP, CA, SP)	ns	ns	ns
					○ K-CBCL An/dep	p = .03	ns	p = .02
					○ K-CBCL In	p = .02	ns	p = .03
		No hearing, visual, or physical disabilities preventing outdoor sport activities No clinically significant physical or neurological illnesses inhibiting treatment.			○ AHWA-VABS socialization	p < .01	p < .01	p < .01
					○ BDI (F, M)	ns	ns	ns
					○ STAI-T (F, M)	ns	ns	ns
					○ STAI-S (M)	p < .01	p = .01	p = .04
					○ STAI-S (F)	ns	ns	ns

ABC, Aberrant Behavior Checklist; AD, Autism Disorder; ADI-R, Autism Diagnostic Interview-Revised; ADOS, Autism Diagnostic Observation Schedule (lc-a, language and communication algorithm; lc-t, language and communication total; rsi-a, reciprocal social interaction algorithm; rsi-t, reciprocal social interaction total); AHWA-VABS, Korean Version of the Vineland Adaptive Behavior Scale); ASC, Adapted Skillstreaming Checklist; ASD, Autism Spectrum Disorder; ASDS, Asperger Syndrome Diagnostic Scale (L, Language; SI, Social Interaction; BP, Behavioral Problems; CA, Cognitive Ability; SP, Sensorimotor Problems); BASC-2-PRS, Behavior Assessment System for Children, Second Edition-Parent Rating Scales; BDI, Beck Depression Inventory (F, Father; M, Mother); CASL, Comprehensive Assessment of Spoken Language; CDI, Child Depression Inventory; CGI, Clinical Global Impressions Scale; Clin dx, clinical diagnosis; CON, Control condition; CSI, Children’s Symptom Inventory; DANVA-2, Diagnostic Analysis of Nonverbal Accuracy-2 (AF, Adult Facial Expression; CF, Child Facial Expression); Excl, exclusion criteria; FQS, Friendship Qualities Scale; HFASD, High-Functioning Autism Spectrum Disorder; INT, Intervention; K-CBCL, Korean Version of the Child Behavior Checklist (An/dep, anxiety/depression; In, Internalizing problems); K-SSRS, Korean Version of the Social Skills Rating System (As, Assertion; Co, Cooperation; Em, Empathy; SC, Self-Control; T, Total score); PDD-BI, Pervasive Developmental Disorders: behavior Inventory; PEI, The Pupil Evaluation Inventory (Wi, Withdrawal; Ag, Agression); PHS, Piers-Harris Self-Concept Scale; PRI, Perceptual Reasoning Index; PSS, Parent Satisfaction Survey; QPQ, Quality of Play Questionnaire (Con, Conflict ; Dis, Disengage; Eng, Engage; Gu, Guest; Ho, Host); SCI, Social Competence Inventory (PSI, Pro-Social Index; SI, Social Initiation Index); SCQ, Social Communication Questionnaire; SKA, Skillstreaming Knowledge Assessment; SRS, Social Responsiveness Scale; SSRS, Social Skills Rating System (As, Assertion; SC, Self-Control; In, Internalizing behavior; Ex, Externalizing behavior); STAI, State and Trait Anxiety Inventory (T, Trait; S, State; M, Mother; F, Father); STAIC, State and Trait Anxiety Inventory for Children (T, Trait; S, State; M, Mother; F, Father); TASSK, Test of Adolescent Social Skills Knowlegde; TASSK-R, Test of Adolescent Social Skills Knowledge-Revised; TOPS-ER, Test of Problem Solving-Elementary Revised; VCI, Verbal Comprehension Index; VIQ, Verbal IQ; I, no covariates are controlled; II, controlled teen’s age, sex, IQ and medication as covariates; III, controlled socioeconomic status, maternal education, and age as well as teen’s age, sex, IQ and medication as covariates.

With respect to limitations of the studies, Reichow, Steiner and Volkmar reported that the studies in their meta-analysis had the same limitations as mentioned before. Additionally, Gillies, Carroll & Loos [24] commented on their meta-analysis [23] with additional potential sources of bias, regarding families that could not be blinded for their condition, the exclusion of participants with intellectual disability and the inclusion of only group-based skills training.

As reported in Table 1, the studies used different outcome measurements. Some used specific intervention-related instruments, others used more general social skills instruments. The difference between the concepts behind each instrument and thus the exact outcome of each of the studies, complicates comparison between them. The use of standardized, internationally used and well validated instruments would facilitate measuring efficacy of SSTs and comparison between studies.

To clarify the effectiveness of SSTs for children with ASD, follow-up measurement is also important [24]. None of the studies reported in Table 1 had follow-up measurements for the treatment as well as for the control groups beyond post-treatment, therefore no information is available on the longer term effects of the SST compared to not receiving SST.

Another important issue, which was not addressed in the evaluated studies, is to find out whether the learned skills generalize to situations in the child’s daily life [24]. To assess this aspect, one should measure social skills from everyday life of the child instead of very specific and discrete behaviors learned at the investigated intervention [13]. The use of multiple informants (e.g., parents and teachers) increases the insight into the extent to which the child uses learned social skills in daily life.

Another aspect understudied so far is the benefit of parental involvement in the SST [24]. The clinical impression is that parental involvement in interventions increases generalization of the learned skills. Parents may remind children to practice and to apply their learned skills in various situations in daily life. This may be especially beneficial to children with ASDs who generally have difficulty to learn and change behavioral patterns. A part of the studies investigated an SST with parental involvement [8,9,16,21,22] but only one study compared an SST with and without parental involvement [15]. However, it was unclear whether the significant differences between the two groups, with respect to skills awareness and motivation were due to parental involvement or to the specificity of the training for ASD, since it compared an SST with parental involvement specifically developed for children with HFA to an SST without parent participation not specifically developed for children with ASD.

The best age when to provide an SST is subject to discussion. In clinical practice, SSTs are provided to younger and older children, adolescents, and adults. In early adolescence and adulthood, lack of social skills can result in peer ridicule and rejection [6]. Therefore, it seems important to offer an SST in preadolescence, to prevent children from experiencing more impairment, distress, and internalizing problems [7,15]. The younger a child masters social and communicational skills and concepts, the earlier he or she can apply these in daily life. If effective, the child’s development may then deviate less than without training. Herbrecht and colleagues [25] have found in their study that the children’s group (8 to 13 years old) benefited more from the training than the adolescents’ group (13 to 19 years old). Although the interventions differed in frequency and duration, they interpreted this as a possibility that children are more receptive to intervention than adolescents, because of a higher ongoing natural maturation effect in children or because the psychopathology in children is probably less chronic yet than in adolescents. However, a child can be too young for SSTs as well, because the attention span must be long enough [25], the didactic presentations must not be overwhelming to allow benefit [9], and the children must be able to read and write their homework.

The current study investigates the efficacy of a comprehensive manualized SST, specifically developed for children with ASDs, in the last two years of primary school. In the Dutch school system, children are typically 10–12 years old in these final years of primary school. The study is designed as an RCT with three conditions: SST consisting of 18 group sessions; SST (same content, frequency and duration) plus parent and teacher involvement (so called SST-PTI); and care-as-usual. The study has aimed for a large sample size (n = 120) and pays particular attention to generalization of learned skills in daily life. Possible improvement is measured with standardized, internationally used instruments for specific behaviors and broader functioning in daily life from a multi-informant perspective. Follow-up measures will add to the knowledge of the efficacy of SSTs on the longer term.

### Research aims and hypothesis

The main aim of the current study is to investigate the efficacy of SST as compared to care-as-usual in 10–12 year old children with ASDs. The efficacy is investigated at several levels: on the level of specific behaviors instructed during the intervention and on the level of general social skills at home and at school. Data are collected from multiple informants: children, parents, teachers, and independent observers. The hypothesis is that children participating in SST show greater improvement on all levels than children in the care as usual condition.

The secondary aim is to investigate the efficacy of an enhanced SST (i.e. SST-PTI, involving parents and teachers) as compared to non-enhanced SST, specifically on the generalization of the learned skills. We expect that generalization increases when parents and teachers learn behavioral therapeutic principles to support the child at home or at school in practicing and implementing social and communicative behaviors.

Additionally, the study aims to investigate factors that possibly influence the effectiveness of SST, searching for information on whether and if so which specific groups of children with ASDs improve more or less with SST.

## Methods/Design

The efficacy of the SST is investigated in an RCT, including two intervention conditions (SST and SST-PTI) and a care-as-usual condition [Efficacy of Social skills Training In Autism (ESTIA)].

Two Dutch child mental health centers participate in the study: Accare University Center for Child and Adolescent Psychiatry (with locations in the cities Groningen and Drachten) and Lentis Jonx Autism Team North-Netherlands (ATN; also with locations in the cities Groningen and Drachten). All four locations have a regional function. Participants have been recruited through the four locations and all four provide training groups.

### Participants

Participants in the study are children with a best estimate clinical DSM-IV-TR diagnosis of ASD, including Autistic disorder, Asperger’s disorder, or PDD-NOS, assigned in a multidisciplinary team including a child psychiatrist and psychologist. The diagnostic procedure consisted of an interview with parents on the current behavior and on developmental history of the child and observation of the child in a standardized, playful situation (most often the ADOS). For inclusion in the study, the Autism Diagnostic Interview-Revised (ADI-R [26]; Dutch version [27]) and the Autism Diagnostic Observation Schedule (ADOS [28]; Dutch version [29]) were administered. Participants had been referred to one of the four participating child mental health care centers. Their clinician advised them to participate in a social skills training. Motivation of the child and parents for training is established. Their IQ is above 80, they are in the last two and a half years of primary education and 10 to 12 years old.

Children with a physical condition that hampers participation or who cannot arrange visiting the child mental health center for the training have been excluded from participation. However, medication or co-morbid disorders were no reason for exclusion.

### Informed consent

**Figure 1:**
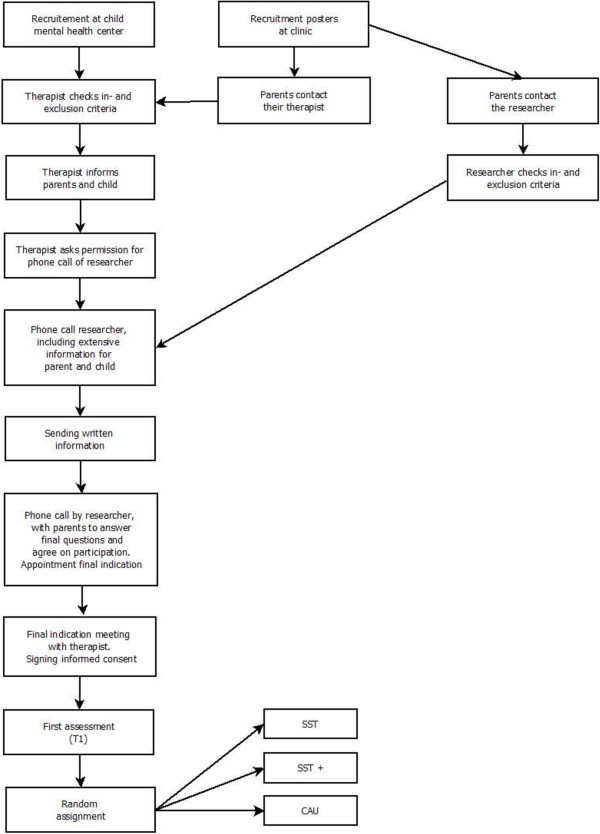
Flow chart of the phases of the RCT.

When an SST was indicated, the therapist introduced this form of treatment to the parents and children and informed them about the study. Sometimes, parents or children were the ones who expressed interest in participating in a social skills training. In that case, the therapist discussed the appropriateness with parents and child and then introduced the study. If parents and children were interested in participating, they agreed that the research team would call them to give more detailed information and would send written information. When the parents and children understood the information from the researchers and were willing to participate, an appointment was scheduled with a therapist. The therapist assessed whether SST was indeed appropriate for the child and the child and parent had the chance to receive more information on the training. If parents, child, and therapist agreed on the appropriateness of treatment and participation in the RCT, the informed consent form was signed by the parents and the child, if aged 12. The researcher informed the teacher and asked for participation after parents had agreed on doing so and the teacher signed informed consent as well. All participants had the right to withdraw from the study at any time, without explanation. Refraining from the study would not affect regular treatment at the child mental health centers. Figure 1 is a flowchart with the phases of the research procedure.

### Therapists

All therapists participating in this study were psychologists who worked at one of the child mental health centers. Two therapists led one group of four to six children. At least one of the (child) psychologists had finished an additional postmaster clinical training. The other had at least a master in Psychology. Both had experience in the broader treatment of children with ASDs and had received training in the theoretical backgrounds and application of cognitive behavioral therapy. Before applying the SST, all therapists participated in a training in the specific protocol. This training lasted six hours for groups of trainers or two hours individually. Trainers received the protocol with all sessions and preparation guidelines. The training focused on the theoretical background, the manualized protocol, the content of the sessions, the physical context of the sessions, the importance of adherence to the protocol, and to the study requirements in general. During the intervention period, therapists had brief meetings or phone calls to discuss the progress of the children or the difficulties in the ongoing groups. These sessions had also been set up to enhance treatment adherence and to prevent therapists to drift from the protocol. In the beginning of the intervention, these meetings were planned weekly, later they were planned once every two weeks. One of the senior therapists of this specific SST supervised these sessions and carefully watched over treatment adherence.

### Randomization

The RCT contains three conditions: SST, SST-PTI, and care-as-usual. As soon as 4–6 children in a treatment center had finished the pretreatment assessment, that group was randomized to one of these three conditions. We used a randomized block design with blocks of 5 with a 2-2-1 ratio (2 SST, 2 SST-PTI, 1 CAU). This balanced randomization procedure was used, striving for unbiased comparison groups, but also for comparison groups of about the same size throughout the trial. The training groups were embedded in three treatment locations that have specific characteristics (Accare Groningen; ATN Groningen; Drachten [including ATN and Accare]). Therefore, we stratified the randomization by the three treatment locations. Research assistants logged in to a web-based program, that allocated groups to treatment condition (randomized on group-level in blocks of 5, stratified per treatment location).

During the inclusion period, the recruiting team was blind to the exact details of the randomization process. To conceal the predictability of the randomization process, research assistants not only entered the treatment location, but also information on the sex of the participants and the number of children in each group (4–6). Sex and group size were in fact not weighed in the randomization.

Randomization took place after all measures of the first assessment were completed. Thus, pre-treatment assessments are independent of the participant’s knowledge of treatment condition.

### Intervention

#### Social skills training

The manualized SST consists of 15 weekly sessions (except for school vacations), followed by three booster sessions starting two months after the 15^th^ session. An SST group consists of four to six children and two therapists. Each session lasts 90 minutes and is video-taped. The SST is based on behavioral therapeutic principles and the social learning theory. The purpose of the SST for children is to learn to interact with other children and to experience that interacting with other children can be fun. Children are taught through instruction, directed positive feedback, observation, and role-play. The therapists analyze the behavior of the children, define individual positive target behaviors, and elicit positive behavior. Negative behavior is ignored when possible, while differentially reinforcing alternative or incompatible positive behavior.

As shown in Table 2, in sessions 1 to 4, the first phase of the SST, the most important aim is to create a safe environment for the children to practice. In sessions 5 to 15, the second phase, nine skills are discussed, practiced, and rehearsed, for instance ‘asking something to someone’, or ‘apologizing to someone’. Sessions 16 to 18 are the so called booster sessions, in which the most relevant skills for each individual child are rehearsed.

**Table 2:** Topics of the social skills training

***Children sessions (in SST and SST-PTI)***		
**1**	Phase 1 (weekly): Create a safe environment	Introduction
**2**		Saying nice things about yourself and to others
**3**		Feelings (showing how you feel and see how another feels)
**4**		Personal presentation (posture, eye contact and use of voice)
**5**	Phase 2 (weekly): Practice skills	Asking something to someone
**6**		Conversation
**7**		Asking for a play date
**8**		Asking to participate
**9**		Discussing with someone
**10**		Playing a social game
**11**		Saying no
**12**		Indicating annoyance
**13**		Apologizing to someone
**14**		Responding to bullying
**15**		Final session, children chose a social activity
**16**	Phase 3 (2 weekly-monthly): Booster sessions	Repeating the above mentioned skills, focusing on individual goals
**17**		Repeating the above mentioned skills, focusing on individual goals
**18**		Repeating the above mentioned skills, focusing on individual goals
***Parents sessions (in SST-PTI)***		
**1**	Phase 1 (weekly): Before child sessions	Psycho-education
**2**		Antecedent interventions
**3**		Consequent interventions
**4 (SST 2)**	Phase 2 (2 weekly): During child sessions	Discrimination training
**5 (SST 4)**		Eliciting desired behaviors and creating opportunities
**6 (SST 6)**		Energizing desired social behavior
**7 (SST 8)**		Responding to and redirecting socially awkward behaviour
**8 (SST 11)**	Phase 3	Continuation and persevere
***Teacher (in SST-PTI)***		
**1**	One meeting with the therapist before the start of the SST for the children en five telephone contacts during the SST (after session 2, 4, 7, 10 and 13).	

Before the start of the training, one of the therapists meets with the child and his/her parents in order to get to know each other and to give practical information. Additionally, this meeting serves as the moment for determining five individual goals of the participant. The parents, child, and therapist together choose five specific behaviors that the child and therapist will focus on during the training.

In order to evaluate the training, the child, parents, and therapists meet and discuss the training and learned skills after the fifteenth session. After the booster sessions, the therapists call the parents for a final evaluation.

For children in this condition, only medication is allowed as an additional treatment.

#### Social skills training – parent & teacher involvement

In the SST-PTI condition, each child follows the exact same procedure as described above for the SST condition. Additionally, his/her parent(s) participate in eight parent sessions. Preferably, both parents participated, however, in many cases only one parent was able or willing to participate. Another addition is that the teacher is coached to support the child in school. An overview of the parent sessions and teacher involvement is presented in Table 2.

The parent sessions are directly linked to the SST sessions and focus on how to support and assist the child in performing the social skills as learned in the training. The sessions consist of instruction on and explanation of behavioral therapeutic principles, behavioral exercises, role-play, and homework. Three parent sessions take place before the children’s SST sessions start. In these sessions, antecedent and consequent interventions are discussed. The parent(s) learn to distinguish desirable social behavior from undesirable social behavior and to elicit the desirable behavior. The other five sessions take place at determined moments during the SST and these are focused on how to support their child in learning and practicing social skills, based on the parental skills from the first three sessions.

Before the children start with their SST sessions, the teacher has one meeting with the therapists concerning (further) education on ASDs, explanation of the SST and specific behavioral instructions. The teachers receive a file with all child sessions and the weekly homework. Additionally, one of the therapists is in contact with the teacher five times throughout the 15 weeks that the child participates in the SST sessions, in order to discuss the possibilities for practicing the weekly homework skills in school and the individual goals.

Also for children in this condition, only medication is allowed as an additional treatment.

#### Care-as-usual

Children in the care-as-usual condition receive no SST. Medication, parent training, and other treatments are allowed and content, frequency, and duration are registered by the researchers. However, parents or child do not receive psychological interventions primarily focusing on improving social skills. Participants in the care-as-usual condition can take part in an SST after the follow-up assessment, one year after start of the SST in the treatment conditions.

### Assessments

Assessments were planned at three moments: before randomization, immediately after the intervention period, and at 6 months follow-up after the end of intervention. The first assessment (T1) was planned after inclusion of the child and the decision of parents and child to participate. This assessment comprises questionnaires for children, parents, and teachers, interviews with parents, and observations of children. Six months later (after SST session 15) the second assessment (T2) was planned, including questionnaires for parents, child, and teacher, an interview with parents, and observations of the child. Another six months later the follow-up (T3) was planned, with all instruments included at T2 for parents and child. No teacher information was going to be collected at this point, since children often have a different teacher on T3 than on T1 and T2 (due to change of class in the end of the school year). In the care as usual condition, T2 was planned six months after T1 and T3 twelve months after T1. Currently, T1 and T2 have been completed. The last group will complete T3 in the summer of 2014.

#### Primary outcome

The primary outcome is adaptive functioning, measured with the Dutch translation of the Vineland Adaptive Behavior Scales - Survey Version (VABS [30]; Dutch Version [31]; measured at T1, T2, and T3), in order to measure the efficacy of the SST in daily life. Adaptive functioning is defined as the performance of daily living activities that are necessary for personal and social functioning of a person [30] and reflects how well children are able to function in daily life. The Vineland measures this competence with three subscales, in the manual called domains: ‘Communication’ , ‘Daily Living Skills’, and ‘Socialization’. It is an open-ended interview with one or both of the parents. Because the SST does not focus on the domains ‘Daily Living Skills’ and ‘Communication’ of the Vineland, only the domain ‘Socialization’ will be used in the analyses.

#### Secondary outcomes parent measures

The Social Skills Rating Scale (SSRS [32]; measured at T1, T2, and T3) is a 38-item standardized parent questionnaire that measures social skills pertaining to home and community settings for children in primary education, completed by the primary caregiver. The subscales included in the analyses are ‘Cooperation’, ‘Assertion’, ‘Responsibility’ and ‘Self-Control’.

The ESTIA - training specific questionnaire (ESTIA-TS; Unpublished manuscript, 2010; measured at T1, T2, and T3) is a 30-item parent-report questionnaire about the specific social skills children learn during the SST. The questionnaire was developed with the aim to investigate change in the specific behaviors as taught in the SST, such as eye contact, recognizing feelings, and apologizing. Parents report on a scale from 0 to 5 how difficult each skill is for their child. They also report on the frequency of each of the skills.

#### Secondary outcome teacher measure

The Teacher version of the Social Skills Rating Scale (SSRS [30]; measured at T1 and T2) has 30-items in three subscales: ‘Cooperation’, ‘Assertion’, and ‘Self-Control’. All three will be included in the analyses.

#### Other measures

Additionally, possible mediators and moderators of treatment outcome will be assessed, e.g. treatment attendance, genetic factors, severity of ASD, intelligence, symptoms of depression, anxiety, attention deficit, hyperactivity, impulsivity, oppositional behaviors, and parental stress.

### Drop-outs

Children in the intervention groups who dropped out after the start of the SST were encouraged to still participate in the assessments. Post-treatment assessment was organized as soon as the child dropped out of treatment, if the drop out was before session 8. For the follow-up assessment the regular schedule has been followed. Children who dropped out after session 8 were invited for the regular post-treatment assessments.

### Sample size calculation

We computed the minimally required sample sizes for two repeated-measures (RM-)ANOVAs on the primary outcome measure VABS, with one between-subject factor (group; 2 levels) and one within-subject factor (time; 2 levels: T3 versus T1); in one test, the SST and care-as-usual groups will be compared and in the other the SST and SST-PTI groups. For each test, we required a power of .99, a significance level of .01, and assumed a correlation between repeated measures of the VABS of .5. Calculations were performed with the program G*Power 3.1.7 [33].

The expected difference between the SST and the care-as-usual group is based on the results of the study into SST of Owens et al. [34], who found a difference between the treated and untreated group of 0.90 SD of the mean VABS outcome. Assuming no effect in the untreated group, the associated effect size f = .4, yielding a minimal sample size of 21 per group. We expect that comparing the two treatment conditions yields lower values of difference. No literature on the comparison of two SST training conditions was available, therefore we defined the difference to detect based on clinical relevance of a difference found. This resulted in a difference between SST and SST-PTI of 0.60 SD of the mean VABS outcome, with associated effect size f = .3, yielding a minimal sample size of 36 per group. Based on our experiences in past research, we presumed a drop-out rate of 10%, implying a minimal sample size of 21/.9 = 24 children in the care-as-usual condition and 36/.9 = 40 in each of the treatment conditions. Because we could include more children in the treatment groups, yielding an even larger power, we aimed 48 in each of the treatment conditions and 24 children in the care-as-usual group (total n = 120).

### Statistical analyses

First, the three groups will be compared with respect to background, age, IQ, and other data in order to assess the comparability between them.

Second, intervention efficacy will be established with hierarchical linear modeling. All data will be analyzed using the intent-to-treat principle. The comparative efficacies of SST, SST-PTI, and care as usual will be investigated with hierarchical linear modeling. The first will test the primary outcome, the socialization domain of the VABS. The other multilevel analyses will be done on each secondary outcome, including 1) parent reported training-specific social skills and 2) more general social skills pertaining to home and community settings, reported by parents and 3) more general social skills pertaining to home and community settings, reported by teacher.

Third, in the completers, the same analyses will be applied, while taking into account possible drop-out and treatment non-adherence. The possible mediators and moderators will be included as explanatory variables into the hierarchical linear models.

### Ethical approval

The Medical Ethical Committee of the University Medical Center in Groningen has assigned ethical approval for the study (METC nr 2009.320).

## Discussion

The current study investigates the efficacy of SST for children with ASDs in the age of 10–12 years. Focusing on the limitations in knowledge so far, the study also investigates generalization of learned skills, long term efficacy, and the influence of parent and teacher involvement in an RCT in a non-US group of 120 children.

### Strengths and limitations

The random assignment of children to one of the treatment conditions or the care-as-usual condition is an important strength of the study. With this design, the two treatment conditions can be compared to the care-as-usual condition and to each other. A detected improvement of efficacy can thus be controlled for development over time. A second important strength of the study is that this is the first study on SST to include a follow-up assessment one year after start of the training in the treated as well as in the control condition. With this follow-up, the long term effect of the SST can be examined. Another strength is the fact that many measures are incorporated in the study, amongst which standardized, international instruments. This may give insight into efficacy at various levels: from specific behaviours and skills to general social functioning in school. Furthermore, if one or both of the interventions show to be effective, the measures may help evaluate why some children improve and others improve less or not at all. Additionally, as recommended in previous studies [6,7,23] the current study has a large sample size (120 participants), in a homogeneous age range, uses a manualized protocol, takes place outside the United States, and involves multiple informants (parents, teacher and child).

A limitation of the study is that only the first assessment could be blinded. During the interventions (or care-as-usual) and at the second and third assessment all therapists, most interviewers, some observers, all children, all parents, and all teachers knew the treatment condition. Future research should aim at blinded interviewers and observers, however, this is very difficult in the setting of the current study as it will be in many other settings.

### Implications for practice

Many parents ask for an SST for their child when they have received an ASD diagnosis and have learned about symptoms and forms of treatment. Additionally, many health care institutions provide SSTs because clinical impression indicates that such a treatment is valuable for a child with ASD. Due to the time-consuming character of SSTs for children, parents, and therapists it is very important to evaluate whether these trainings are effective and if so, for whom.

Additionally, in some of the SSTs parents and teachers are involved on top of the child sessions. In that case, they also invest their time, so information is needed on the added value of their participation for the efficacy of an SST and generalization of learned skills. Before the start of the current study, SSTs were already given in the participating child mental health centers, so the results of this study can be implemented directly.

### Trial status

Participant recruitment started in May 2010 and was finished in September 2013. Currently, in May 2014, all participants have been randomized and completed assessments at T1 and T2. The measurements in the last groups for T3 will take place in summer 2014.

## Competing interests

The authors declare that they have no competing interests.

## Authors’ contributions

VD and AdB drafted the manuscript, EJM, MHN, MET added to and modified the manuscript. All authors read and approved the final manuscript. MHN, EJM and AdB developed the study. MHN, EJM, VD and AdB contributed to the further development of the intervention protocols used in the study, which were based on a protocol initially developed by therapists at Accare. (Barbara van den Hoofdakker, Lianne van der Veen, Sjoukje van Warners, and Leonieke Vet). MET contributed to the statistical underpinning of the study, including sample size calculation and statistical analyses.

## Pre-publication history

The pre-publication history for this paper can be accessed here:

http://www.biomedcentral.com/1471-244X/14/189/prepub
